# Glycosylated haemoglobin (A1c) best values for type 2 diabetes in the battlefield much ado about nothing? (apparently)

**DOI:** 10.1186/s13098-019-0442-x

**Published:** 2019-06-20

**Authors:** Davide Maggi, Fabrizio Montecucco, Gianfranco Adami, Renzo Cordera

**Affiliations:** 1 Diabetes Unit, IRCCS Ospedale Policlinico San Martino Genoa, 10 Largo Benzi, 16132 Genoa, Italy; 2 Department of Internal Medicine, University of Genoa, 6 Viale Benedetto XV, 16132 Genoa, Italy; 3 First Clinic of Internal Medicine, Department of Internal Medicine, and Centre of Excellence for Biomedical Research (CEBR), University of Genoa, 6 Viale Benedetto XV, 16132 Genoa, Italy; 4 IRCCS Ospedale Policlinico San Martino Genoa–Italian Cardiovascular Network, 10 Largo Benzi, 16132 Genoa, Italy

## Abstract

Despite intensive research, therapy of diabetes mellitus type 2 (T2DM) is far from be effective. The most important unresolved issue is to establish a safe glycosylated hemoglobin C (A1c) value well balanced between benefit and side effects. As a result different guidelines suggest different A1c targets generating confusion for patients and clinicians. Here we report two observations which might support a relaxed A1c as suggested by American college of physician (ACP).

## Background

“The prevalence of type 2 diabetes mellitus (T2DM) is rapidly increasing and represents a serious burden for patients and health care systems. Both impaired beta-cell function and insulin resistance contribute to hyperglycemia, the diagnostic hallmark of diabetes. Correction of hyperglycemia is therefore considered a priority of diabetes therapy and it has been pursued with drugs increasing insulin secretion and/or decreasing insulin resistance and obesity, a state of insulin resistance not only simply associated with diabetes but, more important, being a key factors linking T2DM and cardiovascular diseases (CV), the common clinical outcome of diabetes”. This link is so strong that even a modest weight loss is often successful to improve hyperglycemia [1]. Also diabetes remission is observed short time after bariatric surgery before clinical meaningful weight loss [2].

In the last 20 years several clinical trials, designed to correct of hyperglycemia, have been consistently successful to prevent and to decrease microvascular complications of diabetes, while macrovascular complications have been only marginally affected by glucose control.

For many years, insulin, sulfonylureas and biguanides were the only available antidiabetic drugs and their failure to prevent CV diseases has been attributed to the difficulty to lower enough blood glucose concentration due to increased risk of hypoglycemia and increased body weight.

Recently, new classes of antidiabetic drugs with a better clinical profile have become available: it should be recognized however that these new drugs are less prone to cause hypoglycemia but pay different clinical tolls, most of them probably unknown. Unfortunately their anti-hyperglycemic potency is superimposable to that of older drugs and none of them is a disease modifier in term of atherosclerotic complications.

Addressing therapy to glucose control has two main aims: to avoid acute glycemic crisis and to reduce vascular complications. Since vascular complications take years to develop it is not surprising the need long lasting therapy to demonstrate a benefit from a particular strategy or medication.

This means a significant lag-time between glucose control and reduction of vascular complications. As a consequence a patient could suffer from early side effects of therapy without a guarantee of clinical advantages in the long term follow up.

The best glucose control, as A1c to be pursued in a single patient with a balance between early side effects and future benefits is a highly debated issue. Guidelines are not concordant on A1c value, leaving clinician without clears indications and generating concern in patients and doctors. Treatment of hyperglycemia is not free of complications, and might, by itself, lead to increased mortality, poor quality of life, increased cost. A tailored A1c value for each patient should be the best strategy allowing to establish a strict control. Thus “the lower the blood glucose, the lower the risk of microvascular complications” may be unrealistic. As a consequence in the absence of clear guidelines it is not surprising that establishing an A1c target depends on the physicians attitude more than on guidelines themselves and on the identification of those frail patients that never would get advantage from very strict glycemic controls.

It is easy to predict that in the next future choosing a more or less strict glucose control will not be based on evidence based medicine.

In 2018, the American college of physicians (ACP) published revised guidelines for T2DM suggesting to target a 7–8% A1c in most subjects with diabetes. This statement was followed by big criticism and was refused by diabetes and endocrine organizations [3]. It is bitter to admit that the same body of evidence gave rise to quite different clinical advises. One year later, differences between guidelines still persist, with the result of more confusion for both patients and physicians.

One should ask: is there a real clinical difference between HbA1c target values varying of 0.5%? [3]. At first glance a 0.5% lower A1c sound trivial, but in clinical practice means more drugs, more side effects, high cost and increased life burden. The suggestion by ACP, but not ADA, to weaken intensive antidiabetic therapy if HbA1c is 6.5% or lower is suggestive of a different view on the importance of glucose lowering. This debate unfortunately did not determine any change in previous targets, leaving clinicians without clear indications on best HbA1c target.

We propose two further observations that might favor the ACP suggestions for a higher still safe HbA1c target: a different interpretation on early data of an association between glucose control and microvascular complications and the observation in monogenic types of diabetes of the different incidence of microvascular complications, correlated with HbA1c values consistent with the safety of values around 7–8%.

Revisiting Pirart observation, it should be noticed that microvascular diabetic complications were more frequent in patients with higher glycosuria [4]. At that time, these data were considered as a proof that for blood glucose “the lower is better” for patients. Indeed, these results might support another conclusion: glycosuria threshold might represent a threshold glucose plasma concentration above which micro- and macro-vascular complications can be accelerated in the presence of other risk factors. The UK Prospective Diabetes Studies (UKPDS), designed to demonstrate that insulin was better than diet advice for preventing diabetic complications, was unable to help to establish a safe glucose goal. Even if correlations between blood glucose and microvascular complications are linear, some acceleration of progression of microvascular complications was shown for HbA1c values higher than 8–8.5% [5]; Nevertheless a general consensus on a HbA1c lower than 7% as a treatment goal, even if not experimentally proven has become generally recommended [3].

The subtype of monogenic diabetes offer some clues to further clarify the role of blood glucose on development of microvascular complications. patients with MODY 2 (maturity onset diabetes of the young) have a HbA1c between 7–8% lifelong but do not develop significant diabetic complications. On the contrary, patients with other types of MODY, such as MODY 3, have HbA1c higher than 8.5–9.0% lifelong and develop diabetic complications [6].

Medications for blood glucose control in T2DM are associated with risk of significant complications, but with limited experimental evidence of real benefit: HR for death is marginally decreased by lowering HbA1c from 8 to 7% as well as progression of development of microvascular damage [7].

## Conclusion

In our opinion a “safe” HbA1c is that suggested by ACP (7–8%) should be recommended and if new treatments will bring new evidences, they will be more than welcomed. In the mean time a less glucocentric diabetes therapy strategy will allow to implement a more comprehensive multifactorial therapy”.

## Data Availability

Not applicable.

## Abbreviations

A1c: glycated hemoglobin
T2DM: diabetes mellitus type2
CV: cardiovascular
ACP: American college of physician
ADA: American diabetes association
MODY: maturity onset diabetes of the young
UKPDS: United Kingdom prospective diabetes study
